# Supplementary material for: The genome sequence of a gelechiid moth, Gnorimoschema epithymella (Staudinger, 1859) (Lepidoptera: Gelechiidae)
Source: Wellcome Open Res. 2026 Sep 14;11:653. [Version 1] doi: 10.12688/wellcomeopenres.27807.1 (PMC13675626; doi:10.12688/wellcomeopenres.27807.1)
Supplement: Peer review history

# Reviewer report: Reviewer response for version 1

**DOI:** 10.21956/wellcomeopenres.30600.r171911

**Published:** 2026-10-20

**Authors:** James B Whitfield

**Affiliations:** 1 University of Illinois at Urbana-Champaign, Urbana-Champaign, Illinois, USA

This is a nicely prepared and cleanly written report on the genome obtained for Gnorimoschema epithymella, a gelechiid moth. As part of Project Psyche, it perhaps needs no other justification for sequencing, but if there are any special issues that could be addressed with this species by having the genome available, it would be nice for the authors to include that perspective. Otherwise, the introduction was reasonably detailed about the biology of this species and its congeners. It is certainly nice to have genomes available for this relatively diverse and ecvologically important group of Lepidoptera.

 The methods and sequence date are presented quite clearly and fully, so I have nothing to suggest that needs to be added.

Are the datasets clearly presented in a usable and accessible format, and the assembly and annotation available in an appropriate subject-specific repository?

Yes

Are sufficient details of the sequencing and extraction, software used, and materials provided to allow replication by others?

Yes

Are the rationale for sequencing the genome and the species significance clearly described?

Yes

Are the protocols appropriate and is the work technically sound?

Yes

Reviewer Expertise:

Insect diversity, parasitoid wasp systematics and ecology

I confirm that I have read this submission and believe that I have an appropriate level of expertise to confirm that it is of an acceptable scientific standard.

# Reviewer report: Reviewer response for version 1

**DOI:** 10.21956/wellcomeopenres.30600.r169461

**Published:** 2026-10-16

**Authors:** Ricardo Rodriguez-de-la-vega

**Affiliations:** 1 Université Paris-Saclay, Gif-sur-Yvette, France

Comments on Mutanen et al., "The genome sequence of a gelechiid moth..."

 In this Psyche project Genome Note, the authors present two Earth BioGenome Project (EBP) reference-level, haplotype-resolved assemblies of a single female *Gnorimoschema epithymella* subspecies *brunneomaculella* specimen. Sample preparation, sequencing, and assembly followed the state-of-the-art Wellcome Sanger Institute (WSI) Tree of Life Core Laboratory protocols. The assembly was generated using HiFi reads scaffolded with Hi-C data in hifiasm Hi-C phasing mode. The primary assembly was manually curated using mapped Hi-C reads and contains both sex chromosomes. Although no manual curation was performed on the second haplotype, the assembly quality metrics are nearly identical to those of the primary assembly (EBP metric 7.C.Q69 for both), with the sole difference being the BUSCO completeness, which is lower for haplotype 2 due to the absence of sex chromosomes.

 I detected no technical or presentation issues. Beyond being the first genome ever sequenced from the diverse Gnorimoschemini tribe, the highly standardized presentation of *Wellcome Open Research* Genome Notes leaves little room to discuss interesting features of the released genomes. Such features will likely become evident only when researchers begin analyzing the data in a comparative genomics framework. I would like to point out that Merian element M13 is fused with MZ and that the W chromosome, inferred by read coverage, contains dozens of duplicated BUSCOs (lepidoptera_odb10), many of which originate from 13 different Merian elements. Additionally, a large scaffold (CDRNEX010000001.1, ~11 Mb) in the primary assembly has no matches to lepidoptera_odb10.

 For convenience, I am including links to the DToL quality check and Genomes on a Tree page for this specimen/species.

 https://tolqc.cog.sanger.ac.uk/tol/insects/Gnorimoschema_epithymella/

 https://goat.genomehubs.org/record?recordId=2561350&result=taxon&taxonomy=ncbi#Gnorimoschema%20epithymella

Are the datasets clearly presented in a usable and accessible format, and the assembly and annotation available in an appropriate subject-specific repository?

Yes

Are sufficient details of the sequencing and extraction, software used, and materials provided to allow replication by others?

Yes

Are the rationale for sequencing the genome and the species significance clearly described?

Yes

Are the protocols appropriate and is the work technically sound?

Yes

Reviewer Expertise:

Evolutionary Genomics; Bioinformatics

I confirm that I have read this submission and believe that I have an appropriate level of expertise to confirm that it is of an acceptable scientific standard.

# Reviewer report: Reviewer response for version 1

**Published:** 2026-09-14

**Authors:** Automated Benchmarking

**Affiliations:** 1 Wellcome Sanger Institute, Hinxton, UK

As this article is a Genome Note, Automated Benchmarking has been used to determine whether the reported assembly passes three defined checks established by the Earth BioGenome Project: the raw and assembled data are publicly available, the Earth BioGenome Project three-part metric is met, and the BUSCO score passes the stated threshold. For Gnorimoschema epithymella (ilGnoEpit1), all three checks passed. Data available: pass. ENA study PRJEB96966, raw-read accessions ERR15532230 and ERR15551530, and assembly accessions GCA_977012515.1 and GCA_977012825.1 are public. Earth BioGenome Project three-part metric: pass. Haplotype 1 is reported as 7.C.Q69, meeting the 6.C.Q40 reference standard. BUSCO: pass. Haplotype 1, which is proposed as the reference assembly for this species, has 99.4% complete BUSCOs (97.8% single-copy and 1.6% duplicated). BUSCO software version: 6.0.0; lineage dataset: lepidoptera_odb10. It meets the target of greater than 90% complete and single-copy BUSCOs. This decision uses values reported in the Genome Note and public accession records.

Reviewer Expertise:

NA

I confirm that I have read this submission and believe that I have an appropriate level of expertise to confirm that it is of an acceptable scientific standard.
